# Identification and Characterization of an *Aeromonas hydrophila* Oligopeptidase Gene *pepF* Negatively Related to Biofilm Formation

**DOI:** 10.3389/fmicb.2016.01497

**Published:** 2016-09-22

**Authors:** Hechao Du, Maoda Pang, Yuhao Dong, Yafeng Wu, Nannan Wang, Jin Liu, Furqan Awan, Chengping Lu, Yongjie Liu

**Affiliations:** College of Veterinary Medicine, Nanjing Agricultural UniversityNanjing, China

**Keywords:** *Aeromonas hydrophila*, biofilm, transposon mutant library, oligopeptidase F (*pepF*), virulence

## Abstract

Bacterial biofilms are involved in adaptation to complex environments and are responsible for persistent bacterial infections. Biofilm formation is a highly complex process during which multifarious genes work together regularly. In this study, we screened the EZ-Tn5 transposon mutant library to identify genes involved in biofilm formation of *Aeromonas hydrophila*. A total of 24 biofilm-associated genes were identified, the majority of which encoded proteins related to cell structure, transcription and translation, gene regulation, growth and metabolism. The mutant strain TM90, in which a gene encoding oligopeptidase F (*pepF*) was disturbed, showed significant upregulation of biofilm formation compared to the parental strain. The TM90 colony phenotype was smaller, more transparent, and splendent. The adhesive ability of TM90 to HEp-2 cells was significantly increased compared with the parental strain. Fifty percent lethal dose (LD_50_) determinations in zebrafish demonstrated that the enhanced-biofilm mutant TM90 was highly attenuated relative to the wild-type strain. In conclusion, the *pepF* gene is demonstrated for the first time to be a negative factor for biofilm formation and is involved in *A. hydrophila* pathogenicity.

## Introduction

*Aeromonas hydrophila* is a well-known opportunistic pathogen that widely exists in various aquatic environments (Janda and Abbott, 2010). It not only infects fish and other aquatic animals, resulting in huge economic losses in the aquaculture industry, but also causes various diseases in humans, such as skin infections, gastroenteritis, and necrotizing fasciitis. Therefore, this bacterium has attracted extensive attention in recent years (Jiravanichpaisal et al., 2009; Grim et al., 2013). An increasing number of studies on *A. hydrophila* have focused on bacterial pathogenesis, aiming to identify more serviceable virulence factors and potential vaccine candidates (Pridgeon et al., 2013; Wang N. et al., 2013). The biofilm is considered part of the pathogenic activity of various bacteria (Parsek and Singh, 2003; Kishikawa et al., 2013). Due to the ability to attach to visceral organs and many different material surfaces, biofilms have become major causes of persistent infection and drug resistance (Sakimura et al., 2015).

After adhering to the matrix surface, sessile bacteria will produce so-called extracellular polymeric substances (EPSs), which are primarily composed of exopolysaccharides, proteins and extracellular DNA (eDNA) (Sutherland, 2001; Flemming and Wingender, 2010). In contrast to the simplicity of the planktonic phenotype, biofilm formation is an endless cycle and dynamically changing process that is subdivided into four major phenotypic steps: reversible and irreversible adhesion, microcolony formation, growth of the three-dimensional (3D) community, and dispersion (O’Toole et al., 2000). Multitudinous genes play important roles in each phase of biofilm formation. Flagella and pili are involved in surface attachment by facilitating transport (O’Toole and Kolter, 1998; Merino et al., 2014; Qin et al., 2014). Additional surface proteins, such as extracellular matrix binding protein (Embp) (Christner et al., 2010), biofilm-associated protein (Bap) (Merino et al., 2009) and fibronectin-binding proteins (Fnbp) A and B (O’Neill et al., 2008), affect matrix formation. Additionally, both rhamnolipid and type IV pili contribute to the biofilm dispersal of *Pseudomonas aeruginosa* (Pamp and Tolker-Nielsen, 2007). Biofilm formation is controlled by complicated regulatory networks. In *Roseobacter* species, which are a dominant bacterial lineage in the global oceans, biofilm formation is controlled by the quorum sensing (QS) system (Bruhn et al., 2007). In the *Roseobacter* clade *Ruegeria mobilis*, biofilm formation is associated with intracellular concentrations of cyclic dimeric guanosinmonophosphate (c-di-GMP) signaling (D’Alvise et al., 2014). Additionally, the regulation of biofilm formation was affected by varied environmental signals, such as nutrient limitation or elevation, matrix surface, culture conditions, interspecific competition, temperature and pH (Bruhn et al., 2007).

Knowledge of the factors influencing biofilm formation in *A. hydrophila* is limited, although flagella (Merino et al., 2014) and T6SS effector proteins (Sha et al., 2013) are known to contribute to biofilm formation and the development of the mature biofilm structure is known to be influenced by QS signals (Lynch et al., 2002) and the two-component system (TCS) (Kozlova et al., 2012). In this study, to gain a better understanding of the mechanisms underlying biofilm formation, we screened genes involved in biofilm formation using a library of random transposon mutants of *A. hydrophila* NJ-35. We found that oligopeptidase F played a significant negative role in the biofilm formation of *A. hydrophila* and contributed to the virulence of this bacterium.

## Materials and Methods

### Bacterial Strains, Plasmids, and Growth Conditions

The bacterial strains and plasmids used in this study are listed in Supplementary Table S1. The wild-type *A. hydrophila* strain NJ-35 (CGMCC No.8319) was isolated from a diseased crucian carp in 2010, and its whole genome sequence was published in GenBank (accession number: CP006870). *Escherichia coli* strain SM-10, which carries the shuttle plasmid pMMB207, was used for conjugational transfer in the construction of the complementation strains. *A. hydrophila* and *E. coli* were routinely cultured in Luria broth (LB) or on Luria agar (LA) plates at 28 and 37°C, respectively. According to the selection of transposons or plasmids, appropriate antibiotics were added to the culture media at the following concentrations: kanamycin (Kan; 50 mg/ml), chloramphenicol (Cm; 30 mg/ml), and ampicillin (Amp; 100 mg/ml).

### Screening of the Mutant Library

A library containing 1030 random EZ-Tn5 transposon mutants based on *A. hydrophila* strain NJ-35 was previously constructed in our laboratory (unpublished data) preserved with 25% (vol/vol) glycerinum at -70°C.

To identify genes involved in biofilm formation, all mutant clones were screened by crystal violet staining using 96-well plates as described by Stepanovic et al. (2000) with some modifications. Bacteria were grown to the stationary phase, normalized to an optical density of 1.0 at 600 nm (OD_600_) and then diluted 1:1000 in LB medium. Two hundred microliters (200 μl) of this dilution was added to each well of the 96-well plate and then the plates were incubated statically at 28°C. After incubation for 24 h, the medium was discarded and the plates were washed three times with sterile phosphate-buffered saline (PBS) to remove planktonic cells. Subsequently, the surface-combined biofilms were fixed with 99% (vol/vol) methanol for 20 min. After drying at room temperature (25°C), each well was stained with 200 μl of 1% (wt/vol) crystal violet solution for 15 min. Then, the plates were washed with PBS five times to remove unbound dye. To quantify the biofilm biomass, the bound dye was re-dissolved in 200 μl of 95% (vol/vol) ethyl alcohol and the absorbance was measured at 595 nm using a microplate reader (MultiskanGO, Thermo Scientific, USA). Each mutant clone was repeated in eight wells, and the assay was performed in three independent trials. The wild-type strain NJ-35 and LB medium were used as the positive and negative controls, respectively.

### Identification of the Mutant Strain

To ensure the reliability of the mutants, two pairs of primers specific for *A*. *hydrophila* (*iolE*-F/R) and the EZ-Tn5 transposon (*Tn*-F/R) were designed and used for PCR amplification. When the integration sites of the EZ-Tn5 transposon in each biofilm changed, the mutants were identified using thermal asymmetric interlaced PCR (TAIL-PCR), which is an effective genome walking method for the amplification of unknown flanking sequences (Liu et al., 1995). Six specific primer pairs, including the upstream flanking sequence (SP1, SP2, and SP3) and downstream flanking sequence (SP4, SP5, and SP6), were designed according to the EZ-Tn5 sequence to allow the DNA sequence to be amplified from either end of the transposon. After a three-step nested PCR, appropriate DNA products (the length of the gene fragment in the third step was 100 bp larger than that the length in the second step according to the gel electrophoresis) were obtained and sequenced by Suzhou Invitrogen Biological Technology Co., Ltd. The sequence that had an overlapping region with the transposon was selected to determine the insertion site using the EditSeq software. The BLASTN program was used to compare the DNA sequence with the reference strain NJ-35 to confirm the disturbed genes. All of the primers were designed by Primer Premier 5 except for the six degenerate primers (AD1, AD2, AD3, AD4, AD5, and AD6); all of the primers used are listed in Supplementary Table S2. The detailed operation description of the TAIL-PCR is provided in Supplementary Table S3.

### Construction of the Complementation Strain

The complementation of the TM90 mutant strain was constructed with the pMMB207 shuttle plasmid. The DNA fragments, including the *pepF* gene and its putative promoter and terminator region, were amplified using the primer pair *pepF*-C-F/R containing *Eco*R I and *Hin*d III restriction enzyme sites. Following digestion and purification, the target gene was ligated into the pMMB207 vector. The recombinant plasmid pMMB207-*pepF* was first introduced into *E. coli* SM10 by chemical transformation and then transformed into the mutant strain TM90 using bacterial conjugation to generate the complemented strain cTM90::*pepF*. PCR amplification and sequencing were performed to verify the complementation strain.

### Real-Time Quantitative PCR

Real-time quantitative PCR (qRT-PCR) was performed to measure the transcription level of the disrupted *pepF* gene in strains NJ-35, TM-90 and cTM-90::*pepF*. Additionally, the mRNA expression levels of the *pepF* upstream and downstream ORFs were measured to determine whether they were influenced by the disruption of *pepF*. The gene-specific primers used in this assay are listed in Supplementary Table S2. RNA was extracted from logarithmic-phase bacteria using the RNA Isolation Total RNA Extraction Reagent (Vazyme Biotech). Both reverse transcription and cDNA amplification were performed using the One Step qRT-PCR SYBR Green Kit (Vazyme Biotech). All procedures were performed according to the manufacturer’s instructions. The constitutively expressed *recA* gene was used as the reference gene, and the 2^-ΔΔCT^ relative quantification method was used to analyze the mRNA levels (Livak and Schmittgen, 2001). All gene samples were measured three times using the 7300 Real-Time PCR System (Applied Biosystems).

### Sequence Analysis

The nucleotide sequence analyses were performed with the DNAStar Lasergene software and the online BLASTN program^1^. The promoter was predicted using the online Neural Network Promoter Prediction (BDGP) program ^2^. The protein structure analyses were performed with the online ProParam tool^3^. The BLASTP program was used to predict the PepF structural domains and functional sites. The physical and chemical characteristics were analyzed with the online ExPASy ProtParam tool^3^.

### Biofilm Formation Assay

The biofilm formation abilities of the wild-type strain NJ-35, mutant strain TM90 and complemented strain cTM90::*pepF* were examined using the 96-well microtiter plate method described above.

### Confocal Laser Scanning Microscopy (CLSM) Analysis

To further compare the biofilm formation abilities of strains NJ-35, TM90 and cTM90::*pepF*, CLSM was performed for the 3D characterization of the biofilm (Guilbaud et al., 2015). Bacterial cultures in the stationary phase were adjusted to an OD_600_ of 1.0 and diluted 1:1000 in LB medium. Two milliliters (2 ml) of this dilution was added to each well of a 6-well plate that contained a pre-sterilized microscopic glass slide as the substratum for biofilm growth. After incubating the 6-well plate at 28°C for 24 h for biofilm development, the glass slides were carefully washed with PBS to remove the planktonic cells. Then, 20 μl of fluorescein diacetate (FDA; Sigma) was added to each glass slide and incubated out of the light for 20 min. The slide was placed upside down on the confocal laser scanning microscope (CLSM; Zeiss 700) to observe the biofilm using the argon laser. We examined three wells for each bacterial strain, and image stacks were collected from three random points of the biofilms. Sterilized glass slides with fresh LB medium were used as the control group.

### Bacterial Growth Curves

A single clone from each strain was cultured to logarithmic phase at 28°C until the OD_600_ value reached 0.5. The cultures were diluted 1:100 into a beaker flask containing 100 ml of LB medium. Then, the beaker flasks were incubated at 28°C for 24 h with shaking. Every other hour, bacterial growth was examined by monitoring the OD_600_ using a spectrophotometer (BIO-RAD, USA). The plates used for counting were incubated at 28°C. The growth experiments for each strain were repeated three times. The Graphpad Prism 5 software was used for the statistical analysis and to draw the growth curves.

### Motility Assay

The motility assays were performed based on swimming motility and swarming motility as previously described (Rashid and Kornberg, 2000) with some modifications. Briefly, swim plates composed of 1% tryptone, 0.5% NaCl, and 0.3% (wt/vol) agar were inoculated with bacterial clones from an overnight culture grown on LB agar using a sterile toothpick. The plates were incubated at 28°C for 24 h after sealing with medical adhesive tape to prevent dehydration. Using the same method, swarm plates consisting of LB medium with 0.5% (wt/vol) agar were inoculated with a single colony and incubated at 28°C for 24 h. Motility was assessed by measuring the migration diameter of the bacterial cells moving away from the point of inoculation. The motility assay was repeated five times for each strain.

### Adhesion Assay

The adhesion assay was performed using HEp-2 cells as previously described (Tan et al., 2014). The cells were grown in Dulbecco’s modified Eagle’s medium (DMEM) (HyClone, Thermo) containing 10% fetal bovine serum (FBS) at 37°C with 5% CO_2_ for 24 h to obtain monolayer cells in a 24-well microtiter plate. After washing with PBS, each well was inoculated with 500 μl of a bacterial suspension grown to logarithmic phase and diluted to 2 × 10^5^ CFU/ml in minimum essential medium (MEM). The multiplicity of infection (MOI) was 1:1. The plate was centrifuged at 800 × *g* for 10 min and incubated at 37°C with 5% CO_2_ for 2 h to allow cell adhesion. Then, the plate was washed with MEM three times to remove the non-adherent bacteria. After washing, 100 μl aliquots of 1% Triton X-100 were added to each well to lyse the cells with blending for 10 min. To count the adherent bacteria, the bacterial suspension was diluted 10-fold and cultured on LB plates. Each strain was replicated in four wells, and the experiments were performed in triplicate.

### Determination of the Bacterial Median Lethal Dose (LD_50_)

The animal experiment was performed in accordance with the animal welfare standards, was approved by the Ethical Committee for Animal Experiments of Nanjing Agricultural University, China, and complied with the guidelines of the Animal Welfare Council of China. The LD_50_ assays of different strains were performed with a zebrafish model according to the previous study (Pang et al., 2012). Zebrafish were provided by the Pearl River Fishery Research Institute, Chinese Academic of Fishery Science, and the care and maintenance of the zebrafish also followed established protocols. Bacterial strains grown to logarithmic phase were harvested, washed twice, re-suspended in sterile PBS and adjusted to the appropriate concentrations. For each strain, the suspensions were serially diluted 10-fold from 10^1^ to 10^5^ CFU/ml. After acclimating for 3 days, the zebrafish were intraperitoneally injected with 0.02 ml bacterial suspensions. Every ten zebrafish injected with the same CFU was marked as one group and placed in one tank. The tanks were covered with a breathable lid and static cultured at 28°C. Another ten zebrafish were injected with 0.02 ml of sterile PBS as the negative control. The experiments were performed three times, and the Reed and Muench method (Reed and Muench, 1938) was used to calculate the LD_50_ values.

### Statistical Analysis

The statistical analyses in this study were performed using the IBM SPSS Statistics software. The data were expressed as the mean ± standard deviation, and the error bars represent standard deviations of the means in multiple replicate experiments. Significant differences were indicated when the *P*-value was <0.05 according to one-way analysis of variance (ANOVA) with 95% confidence intervals.

## Results

### Screening for Biofilm Mutants

The *A. hydrophila* NJ-35 transposon mutant library containing 1030 random mutants was screened using crystal violet staining to identify mutants with a modified ability to form biofilms. After the initial screening, the individual biofilm abilities of the presumptive biofilm mutants were retested in at least three independent trials. A total of 38 mutant clones were considered to have significant abnormal biofilm formation compared with the parental strain NJ-35, including 20 biofilm-deficient mutants (1.94%, 20/1030) and 18 biofilm-enhanced mutants (1.74%, 18/1030). The transposon insertion sites of the 38 mutant clones were identified by TAIL-PCR followed by sequence analysis and a BLAST search. Among them, 13 clones (TM35, TM687, TM107, TM277, TM383 and TM126, TM38, TM133, TM626, TM869, TM278, TM706, and TM82) were disrupted in the non-coding region and two mutant clones (TM231 and TM435) were disrupted in the same gene. Therefore, a total of 24 genes were confirmed to be associated with *A. hydrophila* biofilm formation and are summarized in **Table 1**. Among the genes identified were 14 biofilm-enhanced and 10 biofilm-deficient genes.

**Table 1:** Characteristics of the biofilm-modified mutants screened by transposon mutant library.

Function group	Mutant	Locus tag^a^	Insertion site/Gene length (bp)^b^	Putative function of the disrupted genes^c^
**Biofilm-deficient mutants**				
Transcription and translation	TM75	U876_11205	644/659	DNA invertase
	TM106	U876_18045	183/3101	Helicase SNF2
	TM231	U876_18040	550/2480	DEAD/DEAH box helicase
	TM435	U876_18040	860/2480	DEAD/DEAH box helicase
Gene regulation	TM22	U876_19630	434/2711	Histidine kinase
	TM771	U876_00315	489/623	LuxR family transcriptional regulator
	TM785	U876_03430	320/630	Transcriptional regulator, ArsR family
Growth and metabolism	TM101	U876_05500	229/1607	Cell division protein DamX
	TM374	U876_18230	211/1822	Protease, collagenase-like protease, PrtC family
	TM898	U876_23005	110/287	PTS ascorbate transporter subunit IIB
	TM765	U876_02165	912/2597	Bifunctional aconitate hydratase
Function unknown	TM28	U876_00770	359/527	Hypothetical protein
	TM29	U876_00295	583/1625	Hypothetical protein
	TM88	U876_22580	340/659	Uncharacterized conserved protein Smg
	TM426	U876_15405	115/301	Hypothetical protein
**Enhanced-biofilm mutants**				
Structure	TM4	U876_21860	1347/3722	MSHA biogenesis protein MshQ
	TM429	U876_21170	126/323	Fimbrial protein
	TM789	U876_00340	858/1190	Membrane protein‘
Metabolism	TM90	U876_20685	547/1898	Oligopeptidase F (PepF)
	TM158	U876_05575	450/1640	Trehalose-6-phosphate hydrolase
	TM303	U876_01635	321/878	Cation transporter/cation eﬄux family
	TM437	U876_03840	456/1580	Phosphodiesterase
	TM715	U876_10760	679/1223	Diguanylate phosphodiesterase
Other functions	TM553	U876_23065	231/674	Hypothetical protein
	TM610	U876_11965	1089/2171	23S rRNA methyltransferase; rlmL

*^a^DNA homologies with the complete genome sequence of the reference strain NJ-35 were evaluated.*

*^b^Insertion sites were identified using TAIL-PCR and sequence analysis.*

*^c^Putative functions were obtained from the NCBI BLAST online server (http://blast.ncbi.nlm.nih.gov/Blast.cg).*

Among the 14 biofilm-enhanced genes, four showed no homology to genes with known functions. The remaining 10 genes could be characterized into three functional categories as follows: (1) three genes were involved in transcription and translation functions, including U876_11205 which encoded a DNA invertase, U876_18045, which encoded a SNF2 helicase, and U876_1804, which encoded a DEAD/DEAH box helicase; (2) three genes possessed regulatory functions, including U876_19630, which encoded a histidine kinase, U876_00315, which encoded a LuxR family transcriptional regulator, and U876_03430, which encoded an ArsR family transcriptional regulator; and (3) four genes were related to growth and metabolism, including U876_05500, which encoded cell division protein DamX, U876_18230, which encoded a collagenase-like protease, U876_23005, which encoded PTS ascorbate transporter subunit IIB, and U876_02165, which encoded a bifunctional aconitate hydratase.

Among the biofilm-enhanced mutants, the disrupted regions identified were involved in bacterial structure and metabolic regulation. U876_21860 encoded biogenesis protein MshQ, U876_21170 encoded a fimbrial protein, and U876_00340 encoded a membrane protein. Five genes were associated with metabolism, including genes encoding oligopeptidase F, trehalose-6-phosphate hydrolase, a cation transporter, a phosphodiesterase and a diguanylate phosphodiesterase. Additionally, genes encoding a hypothetical protein and a 23S rRNA methyltransferase (*rlm*L) were identified.

**FIGURE 1:**
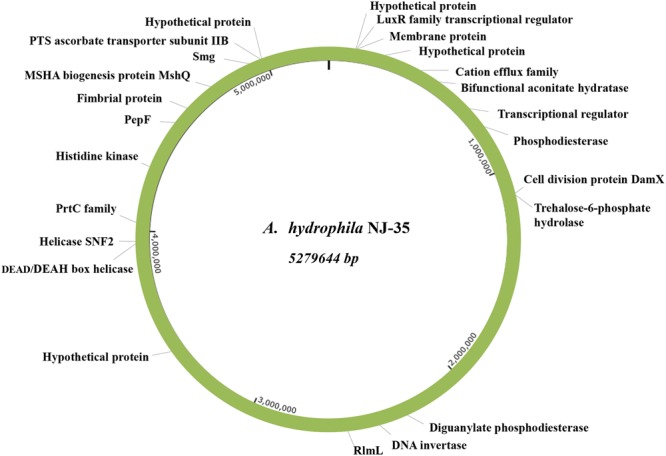
**Relative positions of the identified biofilm-associated genes on the *A. hydrophila* NJ-35 genome map.** Both the biofilm-increased and biofilm-deficient genes are displayed in this map, showing that the EZ-Tn5 transposon was randomly inserted into the genome.

To intuitively show their relative positions, the identified biofilm-associated genes were mapped onto the *A. hydrophila* NJ-35 genome. As shown in **Figure 1**, these disrupted genes were randomly scattered throughout the genome.

### Identification of the TM90 Mutant Strain

**FIGURE 2:**
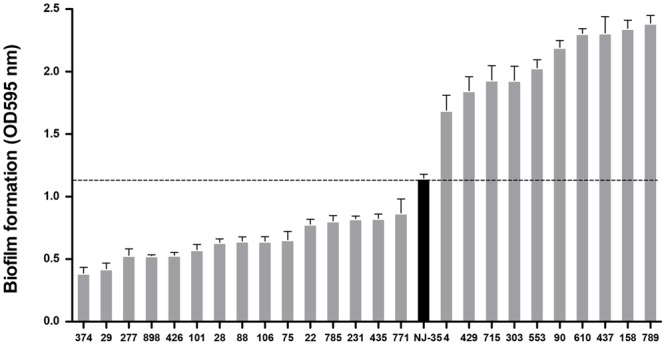
**Biofilm formation of the mutants compared to the wild-type strain NJ-35.** A total of 25 mutants in which transposon insertion sites were identified were ranked in this figure based on their biofilm formation abilities. The black column represents the wild-type strain NJ-35.

As shown in **Figure 2**, the 14 biofilm-deficient mutants showed 24.2–66.2% reductions in biofilm formation, whereas the 10 biofilm-enhanced mutants showed 47.2–108.1% reinforcements in biofilm formation compared with the parental strain NJ-35. Mutant strain TM90, in which *pepF* (U876_20685) was disturbed, showed significant upregulation (91.2%) of biofilm formation compared to the parental strain. The *pepF* gene was considered to be a novel gene involved in biofilm formation, and therefore we selected mutant strain TM90 for further study.

**FIGURE 3:**
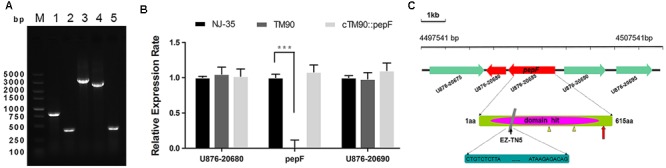
**(A)** Conformation of the *pepF* mutant strain TM90 and its complementation strain. Lane M: DL5000 DNA Marker. Lanes 1 and 2: PCR amplification using primer pairs specific for the EZ-Tn5 transposon (*Tn*-F/R) and the *A*. *hydrophila* strain (*iolE*-F/R) confirmed the reliability of the mutant strain TM90 by showing an 826 bp fragment of EZ-Tn5 and a 401 bp fragment of *iolE*, respectively. Lanes 3–5: The pMMB07 plasmid (3201 bp), *pepF* (2555 bp) and *iolE* (401 bp) were amplified, demonstrating the successful construction of the complementation strain cTM-90::*pepF*. **(B)** The mRNA expression levels of the *pepF* upstream and downstream ORFs and the *pepF* ORF itself in the wild-type strain NJ-35, mutant strain TM90 and complementation strain cTM90::*pepF*. ^∗∗∗^*P* < 0.001, indicates a significant difference in mRNA expression levels for TM90 compared with NJ-35. **(C)** Schematic diagram of EZ-Tn5 transposon insertion in the *pepF* gene. The gene cluster shows the location of the *pepF* gene in the NJ-35 genome. The gray rectangle shows the transposon insertion site in *pepF*, which is located at aa 186. The little yellow triangles indicate catalytic site residues Glu-366 and Tyr-478. The red arrow indicates the presumptive promoter of the upstream gene U876_20690.

To identify mutant strain TM90, PCR amplification using the *iolE*-F/R and *Tn*-F/R primer pairs was performed to confirm the insertion of the EZ-Tn5 transposon into the NJ-35 genome. As shown in **Figure 3A**, Lanes 1 and 2 showed an 826 bp fragment of EZ-Tn5 and a 401 bp fragment of *iolE*, respectively, which confirmed the reliability of mutant strain TM90. The complemented strains were constructed by introduction of the *pepF* gene into the TM90 strain using a complementation vector. Lanes 3–5 showed the pMMB07 plasmid (3201 bp), *pepF* (2555 bp) and *iolE* fragments (401 bp), respectively, demonstrating the successful construction of the cTM-90::*pepF* complementation strain.

The TM90 mutant strain and the complemented strain were identified by PCR with specific primers (**Figure 3A**). The qRT-PCR analysis indicated that pepF mRNA expression was not detected in the TM90 strain, whereas the pepF mRNA was detected by complementation of the mutant strain (**Figure 3B**). Additionally, both the expression of the upstream gene U876_20690 and the downstream gene U876_20680, which encode a chemotaxis protein and hypothetical protein, respectively, showed no significant differences in expression in TM90 compared with NJ-35. This finding indicated that *pepF* inactivation had no polar effect on the transcription of adjacent genes. All of the results demonstrated that the changes in the TM90 mutant strain were directly caused by disruption of *pepF.*

### PepF Characterization

The *pepF* gene encodes oligoendopeptidase F (PepF), which is a metallopeptidase belonging to the M3 family. Members of the M3 family typically contain the HEXXH motif and function in peptide degradation, bioactive neural-peptide synthesis, and cleavage of signal peptides. The nucleotide sequence of the *pepF* gene is 1845 bp in length and encodes a 68.4 kDa protein consisting of 615 amino acid (aa) residues. The amino acid sequence has a theoretical isoelectric point (pI) of 5.42. From an overall perspective, hydrophilic amino acid residues have a larger distribution than hydrophobic amino acid residues. The secondary structure of the PepF protein was predicted to be composed primarily of helices evenly separated by random coils, and only six strands were found throughout the amino acid chain. The conserved domain ranging from aa 160 to 540 was destroyed by transposon EZ-Tn5, which was located at aa 186 in the TM90 mutant strain. To better display the location and structure of the *pepF* gene, a pattern diagram was drawn and shown in **Figure 3C**.

### Biofilm Formation

**FIGURE 4:**
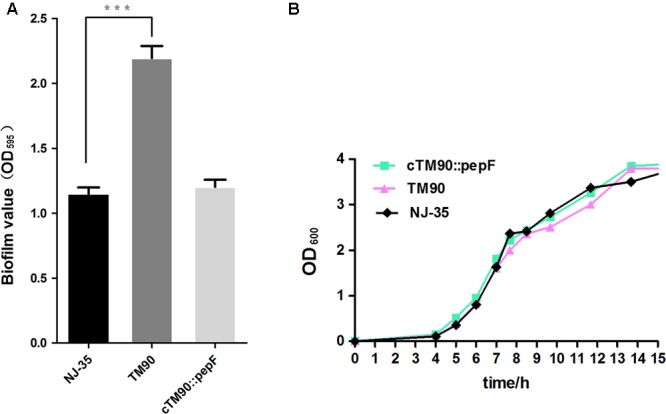
**(A)** Biofilm formation of the wild-type strain NJ-35, mutant strain TM90 and complementation strain cTM90::*pepF*. Biofilm formation was assayed by crystal violet stain using 96-well plates. Data are shown as the mean ± SD of three independent experiments. ^∗∗∗^*P* < 0.001, indicates a significant difference in biofilm formation for TM90 compared with NJ-35. **(B)** Growth curves of the wild-type strain NJ-35, mutant strain TM90 and complementation strain cTM90::*pepF*. The strains were grown in LB medium. The OD_600_ values are the means ± SD from three independent experiments.

**FIGURE 5:**
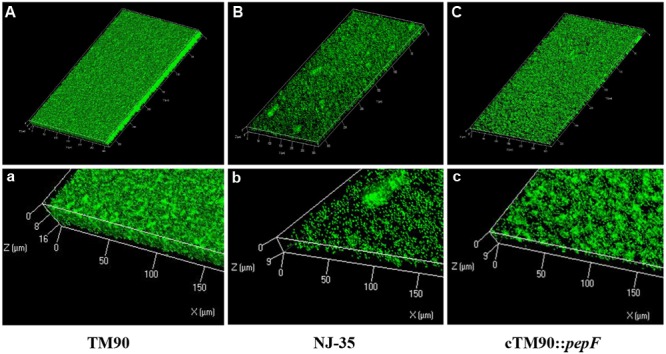
**CLSM images of the 24-hour-old biofilms of the wild-type strain NJ-35, mutant strain TM90 and complementation strain cTM90::*pepF*.** The viable cells presented green fluorescence. **(A–C)** Represent CLSM images of NJ-35, TM90, and cTM90::*pepF*, respectively. **(a–c)** The enlarged areas of the bottom left of the **(A–C)** images and show the local three-dimensional features of NJ-35, TM90, and cTM90::*pepF*, respectively.

The biofilm formation abilities of the wild-type *A. hydrophila* NJ-35 and TM90 mutant strain are shown in **Figure 4A**. The ability of TM90 to form biofilms was significantly increased by 91.2% compared to the wild-type strain (*P* < 0.001), whereas biofilm formation was restored to the wild-type level in the cTM90::*pepF* complemented strain. CLSM of the NJ-35, TM90, and cTM90::*pepF* strains (**Figure 5**) corroborated the results obtained from the crystal violet assay. Mature 24-h-old biofilms were stained with the FDA fluorochrome. Overall, the biofilm formation phenotypes of the NJ-35 (**Figures 5A,a**) and cTM90::*pepF* (**Figures 5C,c**) strains were stronger, whereas TM90 (**Figures 5B,b**) exhibited poor biofilm formation. The data indicated that the *pepF* gene contributed to the decreased biofilm formation of *A. hydrophila*.

### Bacterial Growth and Phenotype Identification

**FIGURE 6:**
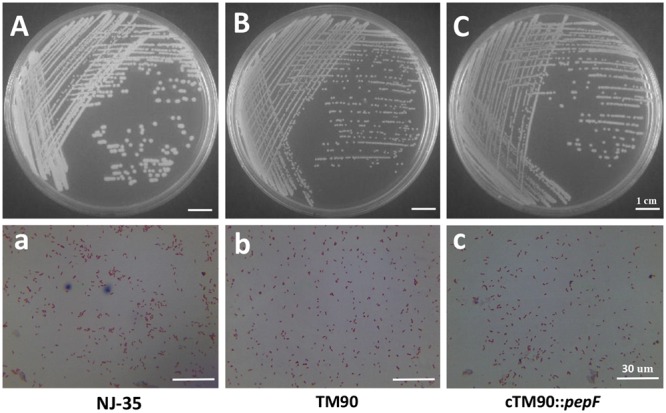
**Colony and microscopic morphology of the wild-type strain NJ-35, mutant strain TM90 and complementation strain cTM90::*pepF*.** The NJ-35 **(A)**, TM90 **(B)**, and cTM90::*pepF* **(C)** colony morphology on the LB plate was observed after culture for 24 h. The microscopic morphology of NJ-35 **(a)**, TM90 **(b)**, and cTM90::*pepF* **(c)** was observed at 100× magnification after Gram staining. The bar represents 1 cm in **(A–C)** and 30 μm in **(a–c)**.

No significant changes in bacterial growth (*P* > 0.05) were detected among the NJ-35, TM90 and cTM90::*pepF* strains when cultured in LB broth for 24 h (**Figure 4B**). However, when cultured on LB plates, the TM90 bacterial colonies (**Figure 6B**) were smaller, more transparent and splendent compared to the NJ-35 strain colonies (**Figure 6A**). Moreover, the TM90 colonies showed higher viscosity when picked with a toothpick. The complemented strain cTM90::*pepF* showed a restored wild-type phenotype (**Figure 6C**), although the growth phenotype was not fully restored. Because TM90 exhibited a dramatically different colony morphology, we examined whether individual mutant cells also differed from the wild-type cells. Under the light microscope, the TM90 cells (**Figure 6b**) were of normal size and shape and were identical to the wild-type (**Figure 6a**) and complemented strain cells (**Figure 6c**).

### Motility Analysis

**FIGURE 7:**
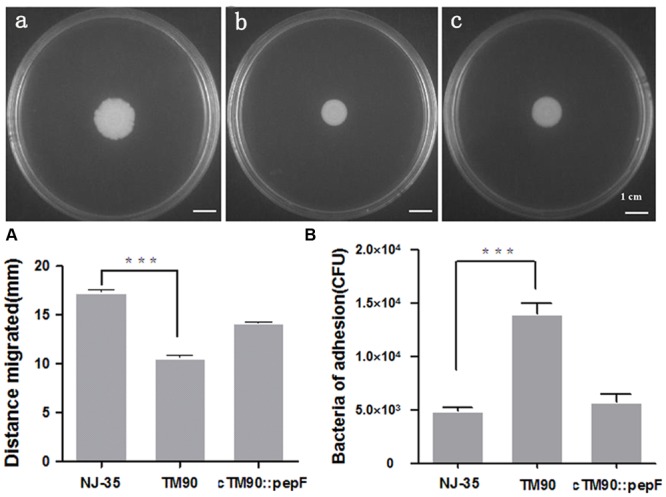
**(A)** Swimming ability of the wild-type strain NJ-35, mutant strain TM90 and complementation strain cTM90::*pepF*. Swimming motility of NJ-35 **(a)**, TM90 **(b)**, and cTM90::*pepF* **(c)** measured by examining the migration of bacteria through the agar from the center toward the periphery of the plate. ^∗∗∗^*P* < 0.001. **(B)** Cell adherence ability of the wild-type strain NJ-35, mutant strain TM90 and complementation strain cTM90::*pepF*. The adherent bacteria from four wells were counted for each strain. The experiment was performed in triplicate. ^∗∗∗^*P* < 0.001.

Swimming and swarming assays were performed to determine the effect of PepF on bacterial motility. Swimming motility of NJ-35 (**Figure 7a**), TM90 (**Figure 7b**), and cTM90::*pepF* (**Figure 7c**) measured by examining the migration of bacteria through the agar from the center toward the periphery of the plate. As shown in **Figure 7A**, migration diameters of 17.7 mm were observed for NJ-35 and 11.0 mm for TM90 on the swimming plates. The motility capacity was partially restored in the complemented strain cTM90::*pepF*. Likewise, the swarming motility showed a change trend similar to the swimming motility, although the differences were not significant (data not shown). The results suggested that the p*epF* gene was involved in *A. hydrophila* motility.

### Adherence Abilities

To determine the effect of PepF on bacterial adherence, the NJ-35, TM90, and cTM90::*pepF* strains were tested on HEp-2 cells. As shown in **Figure 7B**, the adherent TM90 cells totaled 1.4 × 10^4^ CFU/well at an MOI of 1, which was significantly increased compared to the wild-type strain NJ-35 (4.87 × 10^3^ CFU/well) (*P* < 0.001). The bacterial adhesion capacity to HEp-2 cells was partially reduced in the complemented strain cTM90::*pepF.* The results suggested that disruption of *pepF* enhanced the adhesion capacity of *A. hydrophila* to HEp-2 cells.

### Determination of Bacterial Virulence

To determine whether the *pepF* gene affected bacterial virulence, the LD_50_ values of the wild-type, mutant, and complemented strains were measured using the zebrafish model. Typical clinical signs of hemorrhagic septicemia were observed in the dead fish. The LD_50_ values were 1.72 × 10^2^ CFU for the wild-type strain NJ-35 and 1.48 × 10^3^ CFU for the mutant strain TM90. The LD_50_ values of the complemented strain TM90::*pepF* recovered the bacterial virulence (1.83 × 10^2^ CFU). The LD_50_ value of TM90 was increased by 8.6-fold compared with the wild-type strain NJ-35, indicating that TM90 was highly attenuated. This finding suggests that PepF may contribute to *A. hydrophila* virulence.

## Discussion

As a powerful approach for functional gene analysis, DNA transposition has been performed in multifarious bacteria, including *Listeria monocytogenes* (Chang et al., 2012), *Riemerella anatipestifer* (Wang X. et al., 2014), *Pseudomonas putida* (Xia et al., 2015) and *Salmonella typhimurium* (Sahu et al., 2013), to ascertain genetic determinants. In this study, we identified 24 biofilm-associated genes based on a library of 1030 mutant colonies of *A. hydrophila*, among which 14 genes were shown to enhance biofilm formation and 10 were involved in reducing biofilm formation. Another 13 mutant strains associated with biofilm formation had transposon insertions in non-coding regions. The most likely explanation for this finding is that the transposons inserted into gene regulatory regions, such as the basal promoter element, and therefore the functions of the upstream or downstream genes were disrupted, resulting in a change in the biofilm formation ability. Our data provide further proof that biofilm formation is a complicated and highly regulated process.

Of the 24 genes, 11 were previously reported to be associated with biofilm formation. These genes encode histidine kinase (Brooks and Mandel, 2016), LuxR (Wang H. et al., 2014), fimbrial protein (Wang C. et al., 2013), membrane protein (Smith et al., 2016), helicase (Oun et al., 2013; Granato et al., 2016), transcriptional regulator of the ArsR family (Mac Aogain et al., 2012), trehalose-6-phosphate hydrolase (Wu et al., 2011) and phosphodiesterase (Feirer et al., 2015; Jiménez-Fernández et al., 2015). The histidine kinase in combination with the response regulator constitutes a TCS that controls many bacterial pathogenic behaviors, including biofilm formation, virulence, and antibiotic resistance (Wang et al., 2009). Some TCSs can regulate bacterial characteristics and promote a switch from a motile to a sessile bacterial lifestyle, as shown in *P. aeruginosa* (Mikkelsen et al., 2011), via either the production of extracellular appendages such as flagella, type IV pili and Cup fimbria or exopolysaccharides such as Pel and Psl. The LuxI/LuxR QS system plays an important role in communication with members of Gram-negative species (Nealson and Hastings, 1979). The identification of genes known to be associated with biofilm formation indicates the reliability of our screening method for the identification of new genes associated with biofilm formation.

One thing to be noted, in previous studies, fimbria (Wang C. et al., 2013) and membrane protein (Smith et al., 2016) in some bacteria were regarded as the positive factors in biofilm formation. However, in our study, the two proteins were shown to negatively relate to biofilm formation. This phenomenon might be due to bacterial species. Phosphodiesterase was regarded as a positive factor in biofilm formation in *Agrobacterium tumefaciens* (Feirer et al., 2015), whereas in this study and the investigation for *Streptococcus mutans* (Peng et al., 2016), it was a negative factor. Another possible explanation is that different genes of the same functional protein category may contribute to biofilm formation in different ways. Also, a same gene may play different roles in different biofilm formation phases. For example, the type IV pili contributed to multiple roles in structural biofilm development – the initial formation and dispersal (Pamp and Tolker-Nielsen, 2007).

Additionally, we identified 13 biofilm-associated genes that had not been previously reported, among which some genes appeared to make positive contributions to biofilm formation and others made negative contributions. It is noteworthy that the disruption of *pepF* significantly increased *A. hydrophila*’s ability (91.2%) to form biofilms. Oligopeptidase F has been demonstrated to interfere in many important cell functions, including protein turnover and the initiation of sporulation (Vimr et al., 1983; Kanamaru et al., 2002; Kleine et al., 2008). To date, few studies have reported a role for *pepF* in bacterial biofilm formation. Dipeptidyl peptidase IV (DPPIV), which also belongs to the M3 family, was more biologically active in bacterial strains with a high capacity to form biofilms compared to non- or weakly biofilm-producing strains in *Porphyromonas gingivalis* (Clais et al., 2014). In *Streptococcus uberis*, LiaR was shown to negatively regulate biofilm formation through its potential target dipeptidase (Salomaki et al., 2015). However, whether *pepF* is directly or indirectly involved in biofilm formation is unknown and further work needs to be performed.

Analysis of the bacterial growth curves showed that there was no significant difference in growth among the NJ-35, TM90, and cTM90::*pepF* strains, suggesting that the negative role of PepF in biofilm formation was not the result of changes in bacterial growth, as was shown in *R. anatipestifer* (Wang X. et al., 2014). However, the disruption of the *pepF* gene caused an alteration in colony morphology in TM90. Colony morphology is a complex phenotype that is influenced by the ability of cells to interact with one another. A strong correlation between colony morphology and biofilm formation was demonstrated in *Vibrio cholera* (Fong et al., 2006), *P. aeruginosa* (Kirisits et al., 2005), and *Staphylococcus aureus* (Yarwood et al., 2007). The modification of colony morphology may be a sign of altered expression of one or more bacterial traits. The correlation between bacterial features and colony morphology is unknown and remains to be defined in a future study.

*Aeromonas* spp. generally has two distinct flagellar systems: a polar flagellum that mediates swimming in liquid and multiple lateral flagella that perform swarming over surfaces (Kirov et al., 2004). Flagella-mediated swimming motility has been shown to play an important role in biofilm formation by mediating adherence to eukaryotic cell surfaces and abiotic surfaces (Semmler et al., 1999). However, in our study, we found that the TM90 mutant exhibited impairment in motility (especially swimming motility) compared with the wild-type strain NJ-35. One possible explanation is that although flagella-mediated motility is required for the initial attachment of bacteria to a surface, the down-regulation of motility can help bacterial cells stay together in the biofilm, as was shown in *P. aeruginosa* (Barken et al., 2008; Li et al., 2015). Additionally, regulatory mechanisms may vary greatly among different bacterial species, and flagella-mediated swimming may play different roles in the process of dynamic changes in the biofilm.

Published studies have showed that there was a correlation between biofilm formation and adherence to host epithelial cells (Wang Y. et al., 2014). A similar phenomenon was also found in our study. In agreement with the measured increase in biofilm formation in TM90, a more than 4-fold elevated number of adherent bacterial cells were found in the mutant strain compared to the wild-type strain. This finding led to us to speculate that the disruption of *pepF* might upregulate the expression of adhesins in *A. hydrophila.* There are two types of adhesins in *Aeromonas*: filamentous, including flagella and fimbriae, and non-filamentous, including the OMPs, capsule and LPS. Further studies will be performed to evaluate the specific relationship between adhesion and biofilm formation and to determine the type of adhesin affected in TM90.

Numerous studies have shown that biofilm formation contributes to bacterial virulence (Parsek and Singh, 2003; Kishikawa et al., 2013). However, our results indicated that the enhanced-biofilm mutant TM90 was significantly attenuated in zebrafish compared to the wild-type strain NJ-35. Thus, the data from the virulence assay displayed contradictory results with the biofilm formation and adhesion experiments. This phenomenon could be explained by the possibility that the disruption of the *pepF* gene might exhibit other functional deficiencies. For example, *pepF* disruption may interrupt the expression or secretion of other virulence-associated factors and thus attenuate bacterial virulence in zebrafish. Additionally, the biofilm formation capability is not necessarily associated with increased virulence. For instance, van Hoek (2013) reported that biofilm formation in *Francisella* spp. was most likely a key mechanism for environmental survival and persistence rather than virulence. In this regard, it may be of interest to evaluate how *pepF* regulates bacterial virulence in *A. hydrophila*.

Taken together, we identified 24 biofilm-associated genes through screening of the transposon EZ-Tn5 mutant library. For the first time, we demonstrate that the PepF oligopeptidase has a correlation with biofilm formation as a negative factor and plays an important role in *A. hydrophila* virulence. This study will provide novel insights into bacterial pathogenesis and facilitate the development of new control strategies for *A. hydrophila* infection.

## Author Contributions

YL, HD, and MP conceived the study; MP constructed the EZ-Tn5 transposon mutant library; HD, YD, YW, NW, and JL performed the experiments; YL, HD, and FA drafted the paper; CL contributed the materials and provided valuable suggestions.

## Conflict of Interest Statement

The authors declare that the research was conducted in the absence of any commercial or financial relationships that could be construed as a potential conflict of interest.

## Footnotes

1 http://blast.ncbi.nlm.nih.gov/Blast.cgi

2 http://www.fruitfly.org/seq_tools/promoter.html

3 http://web.expasy.org/protparam/
