# Supplementary material for: Identification and Characterization of an Aeromonas hydrophila Oligopeptidase Gene pepF Negatively Related to Biofilm Formation
Source: Front Microbiol. 2016 Sep 22;7:1497. doi: 10.3389/fmicb.2016.01497 (PMC5032638; doi:10.3389/fmicb.2016.01497)
Supplement: Supplementary file 1 [file Table_1.DOC]

Supplementary Material

**Identification and characterization of an *Aeromonas hydrophila* oligopeptidase gene *pepF* negatively** **related to biofilm formation**

Hechao Du, Maoda Pang, Yuhao Dong, Yafeng Wu, Nannan Wang, Jin Liu, Furqan Awan, Chengping Lu, Yongjie Liu^*^

College of Veterinary Medicine, Nanjing Agricultural University, Nanjing, China

*** Correspondence:**

Corresponding author

[liuyongjie@njau.edu.cn](mailto:liuyongjie@njau.edu.cn)

# Supplementary Table

**
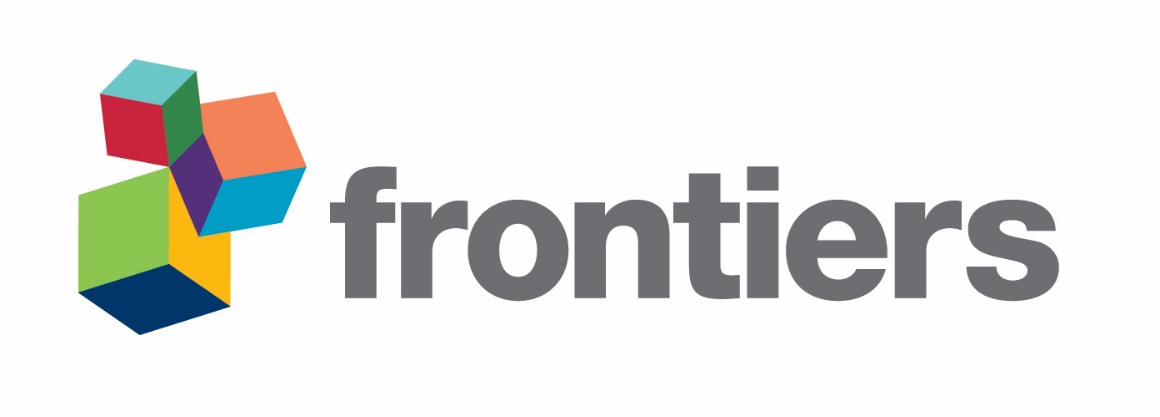
**

**Table S1 Bacterial strains and plasmids used in this study**

| **Strains or plasmids** | **Description** | **Source or reference** |
| --- | --- | --- |
| **Strains**  NJ-35  TM90  cTM90::*pepF*  *E.coli* SM-10  **Plasmids**  pMMB207  pMMB207-*pepF* | A wild-type strain of *A. hydrophila* , isolated from diseased crucian carp; Amp^r^  EZ-Tn5 insertion mutant of *A. hydrophila* NJ-35; *pepF*::Tn  TM90 complemented with the vector pMMB207-*pepF*; Amp^r^, Cm^r^  Production of recombinant plasmids; λpir+, Kan^r^  *E.coli-A. hydrophila* shuttle plasmid; Amp^r^, Cm^r^  pMMB207 carrying *pepF* gene with its putative promoter | This study  This study  This study  (Zhu et al., 2003)  (Morales et al., 1991)  This study |

Morales, V.M., Bäckman, A., and Bagdasarian, M. (1991). A series of wide-host-range low-copy-number vectors that allow direct screening for recombinants. *Gene* 97(1), 39-47. doi:10.1016/0378-1119(91)90007-X

Zhu, J., Chai, Y., Zhong, Z., Li, S., and Winans, S.C. (2003). Agrobacterium bioassay strain for ultrasensitive detection of N-acylhomoserine lactone-type quorum-sensing molecules: detection of autoinducers in Mesorhizobium huakuii. *Applied and Environmental Microbiology* 69(11), 6949-6953. doi: 10.1128/AEM.69.11.6949-6953.2003
