# Supplementary material for: Identification and Characterization of an Aeromonas hydrophila Oligopeptidase Gene pepF Negatively Related to Biofilm Formation
Source: Front Microbiol. 2016 Sep 22;7:1497. doi: 10.3389/fmicb.2016.01497 (PMC5032638; doi:10.3389/fmicb.2016.01497)
Supplement: Supplementary file 2 [file Table_2.DOC]

Supplementary Material

**Identification and characterization of an *Aeromonas hydrophila* oligopeptidase gene *pepF* negatively** **related to biofilm formation**

Hechao Du, Maoda Pang, Yuhao Dong, Yafeng Wu, Nannan Wang, Jin Liu, Furqan Awan, Chengping Lu, Yongjie Liu^*^

College of Veterinary Medicine, Nanjing Agricultural University, Nanjing, China

*** Correspondence:**

Corresponding author

[liuyongjie@njau.edu.cn](mailto:liuyongjie@njau.edu.cn)

# Supplementary Table

**
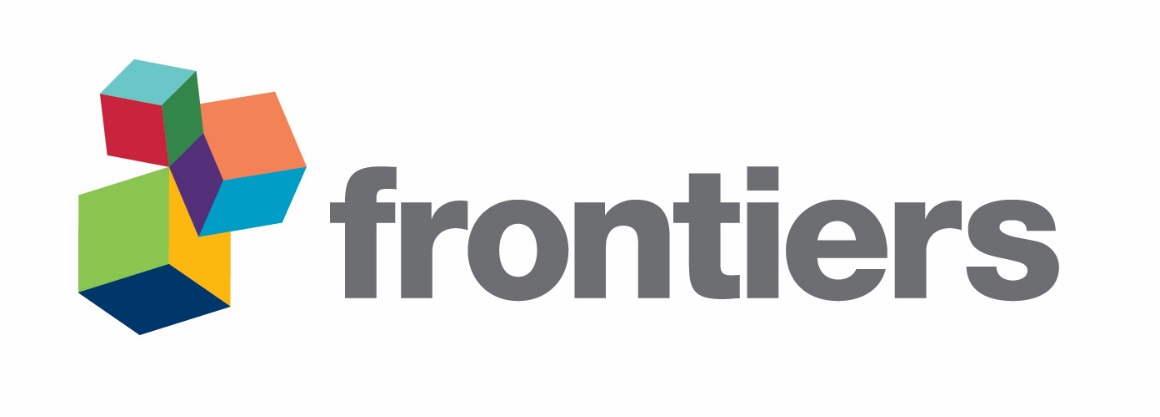
**

**Table S2** Primers used in this study

| **Target gene** | **Length (bp)** | **Primer** | **Sequence (5'-3')** |
| --- | --- | --- | --- |
| ***iolE* ^a^**  **EZ-Tn5**  ***pepF*** | **401**  **826**  **792** | ***iolE*-F**  ***iolE*-R**  ***Tn*-F**  ***Tn*-R**  ***pepF-F***  ***PepF-R*** | **GCAGCGTGGAAGAGGAGAT**  **CTTTGCAGTGAACGTGGTTG**  **GAAACACGGAAACCGAAGACC**  **GCCTGAGCGAGACGAAATACG**  **GCTCGTCCAGCAAGGTCA**  **TCCCGTTCACTCATCAGGTT** |
| **Primers used in Tail-PCR**  **Flanking sequence (upstream) of EZ-Tn5**  **Flanking sequence (downstream) of EZ-Tn5**  **Degenerate primers** |  | **SP1**  **SP2**  **SP3**  **SP4**  **SP5**  **SP6**  **AD1**  **AD2**  **AD3**  **AD4**  **AD5**  **AD6** | **CAGGTTGAACTGCTGATCTTCGGAT**  **TCGGGTGCGGAGAAAGAGGTAA**  **TGCTGCTGGCTACCCTGTGGAA**  **TTGGTTGTAACACTGGCAGAGCATT**  **CGGGACGGCGGCTTTGTTGAATAA**  **CCAACTGGTCCACCTACAACAAAGC**  **(G/C)TTGNTA(G/C)TNCTNTGC**  **NTCGA(G/C)T(A/T)T(G/C)G(A/T)GTT**  **(A/T)GTGNAG(A/T)ANCANAGA**  **NGTCGA(G/C)(A/T)GANA(A/T)GAA**  **TG(A/T)GNAG(G/C)ANCA(G/C)AGA**  **AG(A/T)GNAG(A/T)ANCA(A/T)AGG** |
| **Construction of the *pepF* complemented mutant**  ***pepF-C***  ***pepF*-pMMB207** | **2555**  **3202** | ***pepF*-C-F**  ***PepF*-C-R**  ***pepF*-P-F**  ***pepF*-P-R** | **CCGGAATTCGCCAGTGAGAGCAACAGCA (*Eco*R I)**  **CCCAAGCTTGCCACCGAGAAGAGGATG (*Hin*d III)**  **AACGCCAATCAGCAACGAC**  **ATCCGCCAAAACAGCCAAG** |
| **Primers used in qRT-PCR**  ***pepF-*RT**  ***pepF*-Up-RT**  ***pepF*-Down-RT**  ***recA*-RT** | **116**  **194**  **118**  **182** | ***pepF-*RT-F**  ***pepF-*RT-R**  ***pepF*-Up-F**  ***pepF*-Up-R**  ***pepF*-Down-F**  ***pepF*-Down-R**  ***recA-*RT-F**  ***recA*-RT-R** | **GCACCTTGTAGTCGAAGTAGTC**  **CATGTTCCACACCCTGGAG**  **CTTTGACGGCGACAACTACCTGTTC**  **AGGGGGAGATCCACTTGTATTCCAG**  **CCTGATAGCGGGCATTCTGAT**  **GTGAGCGGTTTTTCGGTGGAT**  **CGACCCCATCTATGCCGC**  **CCATCTCACCTTCGATTTCCG** |

*^a^ iolE* is a specific gene for *A*.*hydrophila*.
