# Supplementary material for: Identification and Characterization of an Aeromonas hydrophila Oligopeptidase Gene pepF Negatively Related to Biofilm Formation
Source: Front Microbiol. 2016 Sep 22;7:1497. doi: 10.3389/fmicb.2016.01497 (PMC5032638; doi:10.3389/fmicb.2016.01497)
Supplement: Supplementary file 3 [file Table_3.DOC]

Supplementary Material

**Identification and characterization of an *Aeromonas hydrophila* oligopeptidase gene *pepF* negatively** **related to biofilm formation**

Hechao Du, Maoda Pang, Yuhao Dong, Yafeng Wu, Nannan Wang, Jin Liu, Furqan Awan, Chengping Lu, Yongjie Liu^*^

College of Veterinary Medicine, Nanjing Agricultural University, Nanjing, China

*** Correspondence:**

Corresponding author

[liuyongjie@njau.edu.cn](mailto:liuyongjie@njau.edu.cn)

# Supplementary Table

**
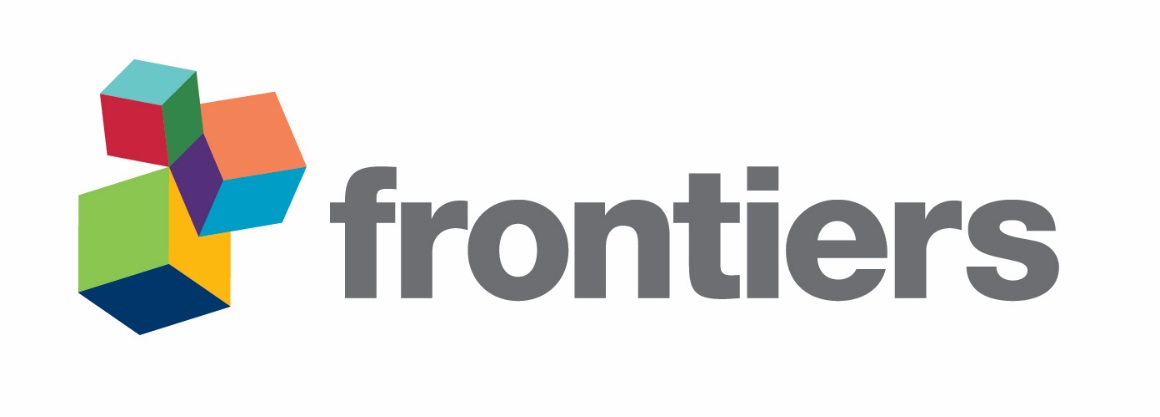
**

**Table S**3 Cycling conditions used for TAIL-PCR

| **Primary cycle of TAIL-PCR** | | | **Secondary cycle of TAILl-PCR** | | | **Tertiary cycle of TAIL-PCR** | | |
| --- | --- | --- | --- | --- | --- | --- | --- | --- |
| **Step** | **Tm(°C)** | **Time(min:s)** | **Step** | **Tm(°C)** | **Time(min:s)** | **Step** | **Tm(°C)** | **Time(min:s)** |
| **1** | **95** | **5:00** | **1** | **95** | **0:15** | **1** | **95** | **0:15** |
| **2** | **95** | **0:15** | **2** | **58** | **0:15** | **2** | **56** | **0:15** |
| **3** | **60** | **0:15** | **3** | **72** | **0:30** | **3** | **72** | **0:30** |
| **4** | **72** | **0:30** | **4** | **95** | **0:10** | **4** | **95** | **0:10** |
| **5** | **go to step 2** | **5 cycles** | **5** | **58** | **0:15** | **5** | **56** | **0:15** |
| **6** | **95** | **0:15** | **6** | **72** | **0:30** | **6** | **72** | **0:30** |
| **7** | **25** | **0:30** | **7** | **95** | **0:15** | **7** | **95** | **0:15** |
| **8** | **ramping to 72** | **0.2°C/s** | **8** | **42** | **0:15** | **8** | **42** | **0:15** |
| **9** | **72** | **2:00** | **9** | **72** | **0:30** | **9** | **72** | **0:30** |
| **10** | **95** | **0:15** | **10** | **go to step 1** | **15 cycles** | **10** | **go to step 1** | **15 cycles** |
| **11** | **60** | **0:15** | **11** | **72** | **10:00** | **11** | **72** | **10:00** |
| **12** | **72** | **0:30** | **12** | **4** | **storage** | **12** | **4** | **storage** |
| **13** | **95** | **0:10** | **13** | **end** |  | **13** | **end** |  |
| **14** | **60** | **0:15** |  |  |  |  |  |  |
| **15** | **72** | **0:30** |  |  |  |  |  |  |
| **16** | **95** | **0:15** |  |  |  |  |  |  |
| **17** | **42** | **0:30** |  |  |  |  |  |  |
| **18** | **72** | **12 cycles** |  |  |  |  |  |  |
| **19** | **go to step 10** | **10:00** |  |  |  |  |  |  |
| **20** | **72** | **storage** |  |  |  |  |  |  |
| **21** | **4** |  |  |  |  |  |  |  |
| **22** | **end** |  |  |  |  |  |  |  |
